# Multimorbidity and mortality thereof, among non-western refugees and family reunification immigrants in Denmark – a register based cohort study

**DOI:** 10.1186/s12889-018-5785-y

**Published:** 2018-07-06

**Authors:** Nasim Taleshan, Jorgen Holm Petersen, Michaela Louise Schioetz, Helle Gybel Juul-Larsen, Marie Norredam

**Affiliations:** 1 Section of immigrant Medicine, Department of Infectious Diseases, Copenhagen University Hospital, Hvidovre, Denmark; 2 Section of Biostatistics, Department of Public Health, University of Copenhagen, Copenhagen, Denmark; 3 Intersectoral Research Unit for Health Services, Bispebjerg University Hospital, Copenhagen, NV Denmark; 4 Research Unit for Chronic Conditions, Bispebjerg University Hospital, Copenhagen, NV Denmark; 5 Optimized Senior Patient Program, Clinical Research Centre, Copenhagen University Hospital, Hvidovre, Denmark; 6 Department of Clinical Medicine, University of Copenhagen, Copenhagen, Denmark; 7 Physical Medicine & Rehabilitation Research-Copenhagen (PMR-C), Copenhagen University Hospital, Hvidovre, Denmark; 8 Department of Physiotherapy, Copenhagen University Hospital, Hvidovre, Denmark; 9 Department of Public Health, Danish Research Centre for Migration, Ethnicity and Health, Section of Health Services Research, University of Copenhagen, Copenhagen, Denmark

**Keywords:** Non-western migrants, Multimorbidity, Secondary health care

## Abstract

**Background:**

The prevalence of multimorbidity, defined by having two or more chronic diseases, is increasing in many Western countries. Simultaneously, the migrant population in Western countries has increased, making up a growing proportion of European populations. This study aims i) to determine the quantity and quality of multimorbidity patterns among refugees and family reunification immigrants from non-Western countries compared to Danish-born, and ii) to compare the mortality burden among those with multimorbidity in the two groups.

**Methods:**

Through the Danish Immigration Service, we conducted a historically prospective cohort study. We identified a total of 101,894 adult migrants who were sub-categorised into refugees and family reunification immigrants, and matched them to a Danish-born comparison group 1:6 on age and sex. Through the Danish National Patient Registry, we obtained information on all in- and outpatient data on hospitalised and ambulatory patients. To assess multimorbidity we used Charlson Comorbidity Index based on ICD-10 codes, together with ICD-10 diagnostic categories for psychiatric disease. We used Cox regression analysis to calculate risk of multimorbidity and risk of mortality in people with multimorbidity.

**Results:**

Overall refugees had higher risk of multimorbidity compared to Danish-born, while family reunification immigrants had a lower risk. When adjusting for civil status and mean income, the risk was lower for all migrant groups compared to Danish-born. Risk of mortality in people with multimorbidity, was lower for all migrant groups, compared to Danish-born.

**Conclusion:**

Refugees are an at-risk group for multimorbidity, however, mortality among those with multimorbidity is lower in all migrant groups compared to Danish-born.

## Background

Multimorbidity can be defined as the co-occurrence of two or more chronic diseases in an individual [1]. A recent study demonstrated that more than half of those with chronic diseases had multimorbidity [2] and that the prevalence of multimorbidity is on the rise in the increasingly aging general European population [3, 4]. Multimorbidity is therefore on the agenda, owing partly to the aging populations and partly to the fact that it challenges existing single-disease modes of delivering health care [5]. Multimorbidity is related to poorer quality of life and functional status together with medication adherence problems, and high mortality, and it entails high healthcare utilization and costs, due to higher rates of hospital admissions and avoidable readmissions [6, 7]. This, together with inadequate clinical practice guidelines for multimorbidity, provides great challenges for health care professionals, including hospital staff and general practitioners [8, 9].

Immigrants make up a growing proportion of the European populations, and in Denmark account for 9.1% of the population by January 1st 2017 [3]. Non-Western immigrants and refugees often come from areas with high prevalence of infectious diseases such as HIV, hepatitis and tuberculosis [10, 11]. Additionally, also diabetes, cardiovascular disease and other chronic diseases are highly prevalent among many non-Western immigrants and refugees, and it is known that some infectious diseases predispose to certain cancer types and diabetes [12–15]. Also, refugee populations are vulnerable to mental distress [7]. Further, accessing health care services is a challenge to some immigrants and refugees due to language barriers, ‘newness’ and cultural differences as well as lack of cultural competent services. This may result in poorer access to and quality of care [16].

Altogether, these factors would hypothetically contribute to more multimorbidity among migrants, however, little is known about the burden hereof among non-Western immigrants and refugees living in Western countries. Previous studies have shown a lower prevalence of multimorbidity, and thus an advantage in health, among refugees and immigrants compared to Western populations [17–21]. However, studies showed that the risk of multimorbidity among immigrants, increased with length of stay in the host country [17, 18, 22]. Still, studies are few and lack: 1) in- and outpatient hospital data; 2) comparisons of different migrant groups with host populations; as well as 3) mortality outcomes among patients with multimorbidity of different ethnic origin. Based on the literature our hypotheses are that we will find less multimorbidity among family reunification immigrants and refugees, compared to native Danes. But, due to the described challenges when meeting the Danish healthcare system, we expect to find higher mortality among immigrants and refugees with multimorbidity. Therefore, our study first aims to determine the quantity and quality of multimorbidity patterns among refugees and family reunification immigrants from non-Western countries compared to Danish-born, and second to compare the mortality burden among those with multimorbidity in the two groups.

## Methods

### Study population

Through the Danish Immigration Service, we conducted a historically prospective cohort study comprising migrants and Danish-born > 17 years of age. Data was provided on all migrants, who obtained right of residency as refugees or through family reunification in Denmark from January 1st 1993 to December 31st 2011. We obtained information on age, sex and nationality and basis for residence permit (refugee vs. family reunification) upon arrival. This amounted to a total of 114,331 migrants. Further, through Statistics Denmark we identified a Danish-born comparison group, matched to the migrants 1:6, on age and sex, on the first day of the year in which the residence permit was given [23]. Next, we excluded migrants who had resided in Denmark for less than 2 years, and the last date to obtain residency was December 31st 2009. We did this to avoid misclassification of people with chronic diseases, considering the time from arrival to registration of disease in a medical facility. We excluded people coming from Western countries incl. The EU (*n* = 8055) because we wanted to focus only on non-Western refugees and family reunification immigrants. This way, we identified a total of 101,894 migrants. The population is divided by migrant status into refugees and family reunified immigrants, and grouped based on region of origin, reflecting the largest regions of origin in the study population: 1) Former Yugoslavia and Eastern Europe; 2) Somalia and Sub-Sahara; 3) Southern and Eastern Asia; 4) Western Asia and North Africa incl. Turkey and Iraq and 5) South America. Although, migrants from South America where left out of the analyses because of their small number. We based our definition of geographical areas on the division by Statistics Denmark.

### Data sources

All persons with permanent residence in Denmark have a unique personal identification number (PIN) which can be used to track them through public registries at an individual level. Using the PIN number, we obtained data on diagnoses via register-linkage to the Danish National Patient Registry (DNPR), which was established in 1977 and represents a key Danish health register. Originally intended for monitoring hospital activities, it now also serves as a source register for more specific registers such as the Danish Cancer Registry and as the basis for the payment of public hospitals via the Diagnostic Related Group (DRG) system. DNPR covers all in- and outpatient admissions to somatic wards. DNPR includes data on date of contact, diagnoses, examinations, and treatment, including operations. The register is generally regarded as possessing good validity and coverage. [24]. Since January 1st 1994, ICD-10 has been used to code diagnoses in the DNPR. To assess multimorbidity we used the 17 chronic diseases in the Charlson Comorbidity Index (CCI) [25] and their corresponding ICD-10 codes [26]. Furthermore, we also wanted to address psychological multimorbidity. Since there is no generally used comorbidity index for chronic psychiatric diseases, we chose, like previous papers [27], to include the following four major ICD-10 diagnostic categories: substance use (F10-F19), schizophrenia and psychosis (F20-F29) affective (F30-F39) and nervous (F40-F49) disorders. We only had access to data from the secondary healthcare sector, because the primary healthcare sector in Denmark has no national register of diagnoses. Thus, our data is on hospitalized patients and ambulatory patients.

We obtained data on vital status and date of death or emigration from the Danish Civil Registration System [28]. Since 1875, it has been mandatory to complete death certificates with a registration of cause of death in any case of death occurring in Denmark. The electronic version of *The Danish Register of Causes of Death* as we use it today began in 1970 [29]. The cause of death is coded in accordance with WHO’s ICD-10. Until 2007, medical officers at the National Board of Health centrally coded causes of death based on information from death certificates. Since 2007, all death certificates have been submitted electronically, and the individual physician does the coding independently.

To consider social status we used data on civil status and income. We used civil status registered at arrival, and used the following 2 categories: Married/Cohabiting and single/divorced/ widowed. Income was based on annual personal income based on wages from earnings and social transfers. Information on income is updated annually on December 31st. We calculated the mean income one year after arrival until one year before first diagnosis. When it comes to migrants, income is a difficult cofounder to adjust for. Migrants will always start at a low income and it will take many years for them as a group to get on the same income level as the background population. To minimize the inequality in annual income, mean income was adjusted for development in wage, which was found to be approximately 2%. We did this to make the migrants more comparable. Personal income was divided into four categories: < 13,400 EURO/year, 13,400–26,800 EURO/year, 26,800-40,200.

### Analysis

**Fig. 1:**
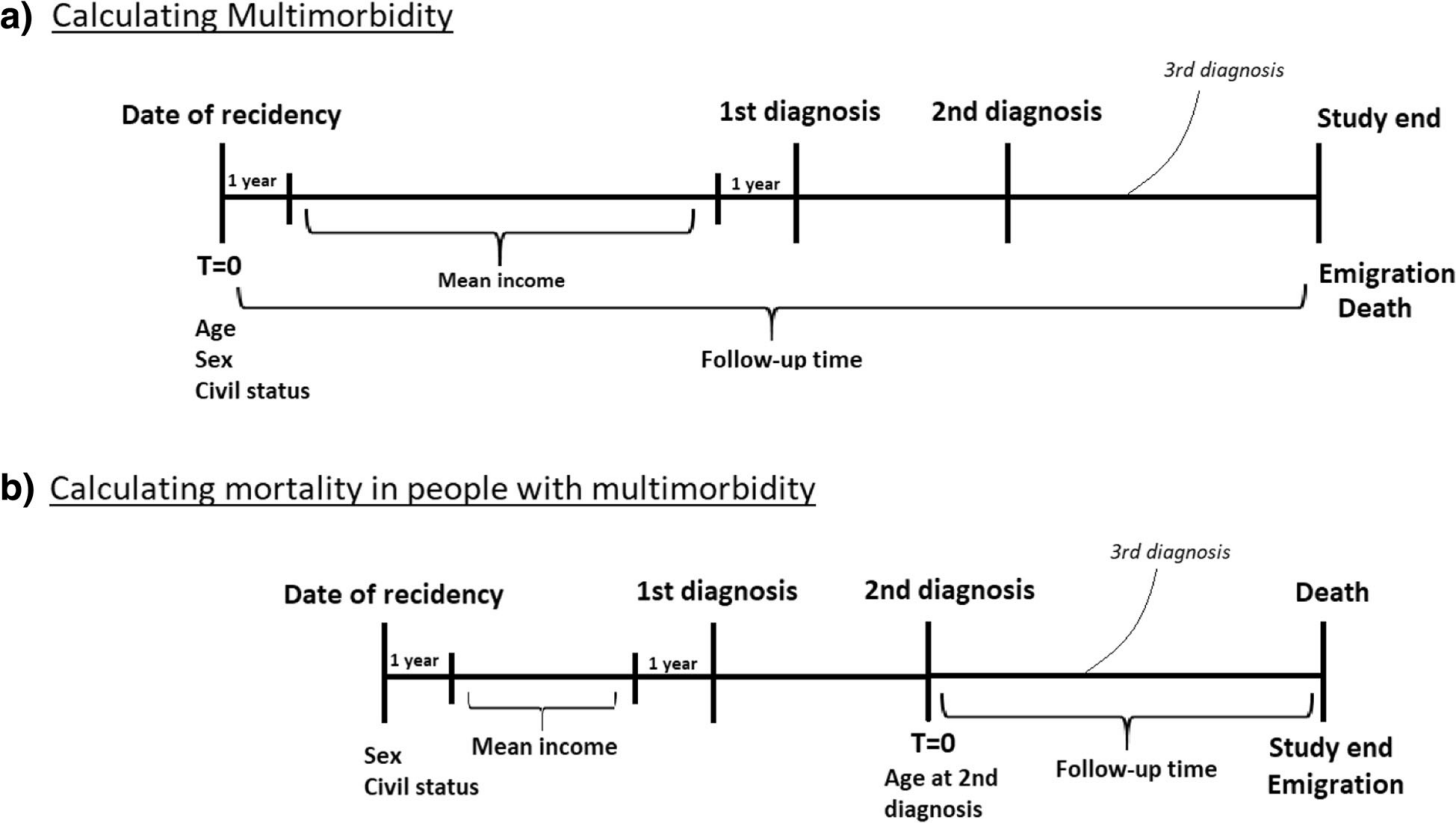
Timeline, depicting how follow-up time and mean income was calculated, and when adjustments were made in **a** calculating multimorbidity and **b** calculating mortality in people with multimorbidity

We calculated Hazard ratios (HR) for multimorbidity using Cox regression analysis. HR were calculated in relation to migrant status and ethnic origin using Danish-born as the reference group. Follow-up was calculated as years from date of residency (for Danish-born, the date of entry of their migrant) until one of the following events: i) first emigration ii) death or iii) study end (December 31st 2011). In this follow-up period, we counted the number of chronic diseases registered for each individual, and only diagnoses registered after the date of entry in the study, were included. Analyses were made joined as well as separately for somatic and psychiatric outcomes. The results were adjusted for dependent variables which were sex, age, civil status and personal income. Differences in mortality among people with multimorbidity were also explored in a Cox regression, adjusting for sex, age, year of 2nd diagnosis, civil status and mean income. Follow-up was calculated as years from 2nd diagnosis until one of the following events: i) first emigration ii) death or iii) study end (December 31st 2011). See Figure 1 for simplification. All statistical analysis was conducted in SAS statistical software version 9.4 (SAS institute, Cary, NC, USA) on the research platform provided by Statistics Denmark. The study was approved by the Danish Data Protection Agency.

## Results

### Population characteristics

We included 101,894 migrants and 611,934 Danish-born control-subjects in this study. The sociodemographic characteristics of these are seen in Table 1. Due to our matching, it is possible to make direct comparison of unadjusted results, that is of “Danish-born matched to refugees” and “Danish-born matched to family reunification immigrants”.

**Table 1:** Sociodemographic characteristics of the migrants (*n* = 101,894) and their Danish-born controls (*n* = 611,934)

	Refugees		Danish-born matched to refugees		Family reunification immigrants		Danish-born matched to family reunification immigrants	
		%		%		%		%
Total (n)	42,696	100	256,452	100	59,198	100	355,482	100
Females (n)	17,903	41.9	107,556	41.9	42,227	71.3	253,487	71.3
Median age at study start (years)	33.0		33.0		28.0		28.0	
Civil status (n)								
Married/Cohabiting	28,167	66.0	134,081	52.3	40,946	69.2	161,759	45.5
Single/divorced/ widowed	11,924	27.9	122,354	47.7	10,356	17.5	193,661	54.5
Missing	2605	6.1	17	0	7896	13.3	62	0
Family income level (euros)								
< 13,400	10,388	24.3	11,460	4.5	9043	15.3	16,722	4.7
13,400 - 26,800	29,115	68.2	129,578	50.5	35,196	59.5	179,114	50.4
26,800 - 40,200	745	1.8	81,775	31.9	6894	11.6	111,815	31.4
> 40,200	56	0.1	17,363	6.8	1023	1.7	20,923	5.9
Missing	2392	5.6	16,276	6.3	7042	11.9	26,908	7.6
Region of origin (n)								
Former Yugoslavia and Eastern Europe	17,931	42.0			11,669	19.7		
Somalia and Sub-Sahara	6598	15.5			6597	11.1		
Southern and Eastern Asia	7169	16.8			19,563	33.0		
Western Asia and North Africa incl. Turkey and Iraq	10,954	25.7			17,790	30.1		
South America	44	0.1			3579	6.0		
Top 5 Countries of origin (n)								
Bosnia-Hercegovina	13,874	32.5			794	1.3		
Iraq	8222	19.3			3556	6.0		
Turkey	60	0.1			7938	13.4		
Somalia	5195	12.2			2674	4.5		
Thailand	0	0.0			5135	8.7		
Median Follow-up (years)	13.4		14.0		10.5		11.2	
End of follow-up (n)								
Emigrations to any country	5454	12.8	13,222	5.2	9678	16.3	20,520	5.8
Death	2223	5.2	24,723	9.6	830	1.4	13,001	3.6
Population at study end	35,019	82.0	218,507	85.2	48,690	82.3	321,961	90.6

Table 2 provides an overview of the distribution of the 17 CCI diagnoses, and the four psychiatric diagnostic groups, and the distribution of multimorbidity among refugees, family reunification immigrants and Danish-born. Overall, chronic diseases and multimorbidity are most frequent in the refugee-group, while it is least frequent in the family reunification group. Both refugees and family reunification immigrants are mostly affected by diabetes, while the Danish-born population is most affected by cancer and chronic pulmonary disease. Of the psychiatric diagnoses, all migrants suffer mostly from nervous disorders, while substance use is most frequent in the Danish groups.

**Table 2:** Distribution of CCI diagnoses, major ICD-10 psychiatric disorders and multimorbidity

Charlson comorbidity index (CCI) diagnoses	Refugees		Danish-born matched to refugees		Family reunification immigrants		Danish-born matched to family reunification immigrants	
	n	%	n	%	n	%	n	%
Acute myocardial infarction	1118	2.6	4159	1.6	342	0.6	2229	0.6
Congestive heart Failure	692	1.6	3436	1.3	278	0.5	1945	0.5
Peripheral vascular disease	331	0.8	2250	0.9	104	0.2	1404	0.4
Cerebrovascular disease	1192	2.8	7675	3.0	619	1.0	5517	1.6
Dementia	137	0.3	1199	0.5	70	0.1	733	0.2
Chronic pulmonary disease	1877	4.4	9870	3.8	1538	2.6	10,748	3.0
Connective tissue disease	352	0.8	2326	0.9	307	0.5	2660	0.7
Peptic ulcer	1960	4.6	3861	1.5	948	1.6	2861	0.8
Liver disease (mild)	150	0.4	1206	0.5	96	0.2	749	0.2
Diabetes	2648	6.2	7005	2.7	1222	2.1	5813	1.6
Diabetes complications	494	1.2	1621	0.6	172	0.3	1249	0.4
Paraplegia/hemiplegia	79	0.2	384	0.1	40	0.1	365	0.1
Renal disease	387	0.9	1781	0.7	201	0.3	1394	0.4
Cancer	1655	3.9	12,657	4.9	922	1.6	10,097	2.8
Metastatic cancer	376	0.9	2891	1.1	168	0.3	1922	0.5
Moderate or severe liver disease	0	0	0	0	0	0	0	0
HIV/AIDS	196	0.5	314	0.1	492	0.8	241	0.1
*1 CCI diagnose*	6357	14.9	28,321	11.0	4775	8.1	28,446	8.0
Multimorbidity (>1 CCI diagnose)								
2 diagnoses	1808	4.2	8841	3.4	854	1.4	6241	1.8
3 diagnoses	616	1.4	3026	1.2	184	0.3	1670	0.5
>3 diagnoses	399	0.9	1692	0.7	105	0.2	886	0.2
*Multimorbidity (> 1 CCI diagnose) in total*	2823	6.6	13,559	5.3	1143	1.9	8797	2.5
Psychiatric diagnoses								
Substance use (F10- F19)	807	1.9	9283	3.6	682	1.2	9258	2.6
Schizophrenia and psychosis (F20-F29)	218	0.5	918	0.4	101	0.2	1001	0.3
Affective disorders (F30-F39)	815	1.9	2862	1.1	626	1.1	3653	1.0
Nervous disorders (F40-F49)	1127	2.6	2597	1.0	803	1.4	4015	1.1
*1 CCI or psychiatric diagnose*	7278	17.0	32,526	12.7	5733	9.7	35,712	10.0
Multimorbidity (> 1 CCI or psychiatric diagnose)								
2 diagnoses	2275	5.3	10,874	4.2	1233	2.1	8776	2.5
3 diagnoses	803	1.9	4045	1.6	295	0.5	2663	0.7
>3 diagnoses	515	1.2	2615	1.0	141	0.2	1457	0.4
*Multimorbidity (> 1 CCI or psychiatric diagnose) in total*	3593	8.4	17,534	6.8	1669	3.0	12,896	3.6

The diseases were grouped according to individual disease entities and multimorbidity, differentiating between somatic multimorbidity and combined somatic and psychiatric multimorbidity

### Incidence of multimorbidity

Our results showed that all family reunification immigrants had significantly lower risk of developing combined somatic and psychiatric multimorbidity compared to the Danish-born controls (Table 3). This advantage remained after adjusting for age, gender, civil status and personal income [adjusted HR (CI 95%) = 0.79 (0.74–0.84), *p* < 0.05]. For refugees, crude HR showed a higher risk [HR (CI 95%) = 1.29 (1.25–1.34), p < 0.05], while adjusting for civil status and personal income resulted in a borderline lower risk [adjusted HR (CI 95%) = 0.95 (0.92–0.99), *p* < 0.05]. Variations were seen between the different regions of origin, showing that refugees from Southern and Eastern Asia, and Western Asia and North Africa incl. Turkey and Iraq, had higher risk of combined multimorbidity than Danish-born, even after adjusting for civil status and mean income. The same was true for family reunification immigrants from Somalia and Sub-Sahara. For somatic multimorbidity only a similar pattern was found, with HR becoming significantly lower for refugees and family reunification immigrants, when adjusting for civil status and mean income.

**Table 3:** Crude and adjusted Hazard ratios of having multimorbidity (> 1 diagnose)

	Somatic and psychiatric multimorbidity (CCI + psychiatric diagnoses)			Somatic multimorbidity (only CCI)		
	Crude HR (95% CI)	Adjusted HR^1^ (95% CI)	Adjusted HR^2^ (95% CI)	Crude HR (95% CI)	Adjusted HR^1^ (95% CI)	Adjusted HR^2^ (95% CI)
Native Danes (ref)	1.00	1.00	1.00	1.00	1.00	1.00
Refugees	1.29* (1.25-1.34)	1.20* (1.16-1.24)	0.95* (0.92-0.99)	1.31* (1.26-1.36)	1.20* (1.15-1.25)	0.97 (0.93–1,02)
Former Yugoslavia and Eastern Europe	1.38* (1.33–1.47)	1.09* (1.04-1.14)	0.89* (0.85-0.94)	1.49* (1.41-1.57)	1.12* (1.06-1.18)	0.92* (0.87-0.97)
Somalia and Sub-Sahara	0.71* (0.64–0.80)	1.04 (0.93–1.17)	0.75* (0.66-0.84)	0.72* (0.64-0.82)	1.15* (1.01-1.31)	0.85* (0.75-0.97)
Southern and Eastern Asia	1.33* (1.21–1.47)	1.36* (1.23-1.49)	1.04 (0.95–1.15)	1.22* (1.09-1.36)	1.25* (1.12-1.40)	0.99 (0.88–1.12)
Western Asia and North Africa incl. Turkey and Iraq	1.43* (1.34–1.53)	1.51* (1.42-1.62)	1.18* (1.10-1.26)	1.36* (1.26-1.47)	1.46* (1.35-1.58)	1.18* (1.08-1.27)
Family-reunified immigrants	0.86* (0.82–0.91)	0.85* (0.81-0.89)	0.79* (0.74-0.84)	0.86* (0.81-0.92)	0.85* (0.80-0.90)	0.78* (0.72-0.84)
Former Yugoslavia and Eastern Europe	0.78* (0.70–0.88)	0.69* (0.61-0.77)	0.64* (0.56-0.72)	0.79* (0.68-0.90)	0.68* (0.59-0.78)	0.61* (0.53-0.71)
Somalia and Sub-Sahara	0.97 (0.84–1.11)	1.10 (0.96–1.26)	1.04 (0.90–1.20)	1.04 (0.89–1.22)	1.24* (1.05-1.45)	1.16 (0.98–1.37)
Southern and Eastern Asia	0.86* (0.79–0.94)	0.74* (0.68-0.81)	0.68* (0.62-0.75)	0.89* (0.81-0.99)	0.73* (0.66-0.81)	0.68* (0.61-0.76)
Western Asia and North Africa incl. Turkey and Iraq	0.89* (0.82–0.96)	1.03 (0.94–1.11)	0.94 (0.86–1.03)	0.85* (0.77-0.94)	1.02 (0.92–1.13)	0.93 (0.83–1.04)

*P < 0.05,

^1^Adjusted for age and gender, ^2^Adjusted for age, gender, civil status and mean income

### Multimorbidity patterns

When looking at the pattern of diseases among the groups, we found that the most common dyads among refugees where metastatic cancer and diabetes, among family reunification immigrants it was metastatic cancer and nervous disorders, and in the Danish control groups it was metastatic cancer and chronic pulmonary disease. When looking at the most common triads, we found a combination of acute myocardial infarction, congestive heart failure and diabetes among the refugees, while the most common triad pattern in the other three groups was entirely psychiatric consisting of substance use, affective disorders and nervous disorders. The most common quadrat pattern was the same for refugees and family reunification immigrants, and consisted of acute myocardial infarction, congestive heart failure, diabetes and diabetes complications. For the controls matched to refugees we found a combination of acute myocardial infarction, congestive heart failure, chronic pulmonary disease and peripheral vascular disease, and for controls matched to family reunification immigrants we found a mainly psychiatric pattern of substance use, affective disorders, nervous disorders and chronic pulmonary disease (data not shown).

### Multimorbidity-associated mortality

When calculating risk of mortality in people with multimorbidity we found a significantly lower risk among both refugees and family reunification immigrants compared to Danish-born (Table 4). This advantage was seen in all groups, and both in people with combined somatic and psychiatric multimorbidity and for somatic and psychiatric multimorbidity alone. The calculated risk of mortality in people with psychiatric multimorbidity for family reunified immigrants from Western Asia and North Africa, incl. Turkey and Iraq, [adjusted HR (95% CI) = 0.12 (0.02–0.83), *p* < 0.05)] seems dramatic. This result reflects that, in this group there were 65 with only psychiatric multimorbidity and of them, one died. For Danish-born the same number is 285 out of 1836. See Table 4 for details. Death among people with multimorbidity was related to being male, unmarried/single, low mean income and high age (60–70 years). Mortality related to psychiatric multimorbidity was twice as high for men vs. women in the family reunification group.

**Table 4:** Crude and adjusted hazard ratios for mortality among people with multimorbidity

Migrant status	Mortality among people with multimorbidity (CCI + psychiatric diagnoses)		Mortality among people with only somatic diagnoses (CCI)		Mortality among people with only psychiatric diagnoses	
	Crude HR (95% CI)	Adjusted HR^1^ (95% CI)	Crude HR (95% CI)	Adjusted HR^1^ (95% CI)	Crude HR (95% CI)	Adjusted HR^1^ (95% CI)
Danish born (ref)	1.00	1.00	1.00	1.00	1.00	1.00
Refugees	0.56*(0.52-0.59)	0.50*(0.46-0.53)	0.59*(0.55-0.63)	0.52*(0.49-0.56)	0.31*(0.22-0.46)	0.34*(0.23-0.50)
Former Yugoslavia and Eastern Europe	0.73*(0.68–0.78)	0.59*(0.56-0.64)	0.74*(0.69-0.80)	0.61*(0.57-0.67)	0.31*(0.17-0.54)	0.29*(0.16-0.52)
Somalia and Sub-Sahara	0.40*(0.32–0.51)	0.43*(0.33-0.55)	0.41*(0.32-0.56)	0.43*(0.33-0.56)	0.22*(0.06-0.88)	0.28 (0.07–1.14)
Southern and Eastern Asia	0.36*(0.23–0.44)	0.35*(0.28-0.44)	0.41*(0.33-0.51)	0.39*(0.31-0.49)	0.43*(0.20-0.91)	0.39*(0.18-0.83)
Western Asia and North Africa incl. Turkey and Iraq	0.37*(0.32–0.42)	0.36*(0.31-0.41)	0.39*(0.34-0.46)	0.37*(0.32-0.44)	0.29*(0.15-0.59)	0.41*(0.20-0.83)
Family-reunified immigrants	0.51*(0.45–0.57)	0.39*(0.34-0.45)	0.53*(0.47-0.60)	0.40*(0.35-0.46)	0.36*(0.20-0.63)	0.53*(0.28-0.98)
Former Yugoslavia and Eastern Europe	0.80*(0.65–0.99)	0.59*(0.47-0.74)	0.86 (0.69–1.06)	0.60*(0.48-0.76)	0.50 (0.19–1.34)	0.99 (0.36–2.72)
Somalia and Sub-Sahara	0.48*(0.35–0.66)	0.57*(0.41-0.80)	0.46*(0.33-0.65)	0.57*(0.40-0.81)	0.29 (0.04–2.07)	0.67 (0.09–4.87)
Southern and Eastern Asia	0.44*(0.35–0.54)	0.28*(0.23-0.35)	0.45*(0.37-0.56)	0.29*(0.23-0.36)	0.53 (0.22–1.29)	0.68 (0.27–1.70)
Western Asia and North Africa incl. Turkey and Iraq	0.44*(0.36–0.53)	0.35*(0.28-0.44)	0.47*(0.39-0.58)	0.36*(0.29-0.45)	0.09*(0.01-0.65)	0.12*(0.02-0.83)

*P < 0.05

^1^Adjusted for age at 2nd diagnosis, gender, civil status and mean income

## Discussion

### Multimorbidity

First, we found that multimorbidity was most frequent in the refugee group, while it was least frequent in the family reunification group. When using Cox regression to calculate the risk of multimorbidity among the groups, crude HR showed higher risk among refugees compared to Danish-born, while family reunification immigrants had a lower risk. When adjusting for civil status and mean income, the higher risk among refugees became insignificant, and we found lower risk of multimorbidity among both refugees and family reunification immigrants, compared to Danish-born. Other studies have also found that non-Western migrants have lower risk of multimorbidity compared to Western populations. A study published in 2015 by *Diaz* et al. [21] shows lower probability of multimorbidity among non-Western migrants compared to Norwegian-born, and in 2016 *Gimeno-Feliu* et al. [17] found lower prevalence of multimorbidity among non-Western migrants compared to Spaniards. The study by *Diaz* et al. [21] also compared disease patterns and found many similarities among Norwegian-born and migrants, showing a mental disease pattern among most groups. When depicting the pattern of multimorbidity, we saw that especially metastatic cancer is highly prevalent, and is part of the most common disease-dyads in all groups. Overall the patterns of disease among the groups seem similar. The high prevalence of metastatic cancer in all groups reflects the source of the data, since these are data from hospitalized and ambulatory patients. Furthermore, since we used the CCI, more serious diseases are depicted in these patterns.

### Multimorbidity-associated mortality

Second, we found that, all groups had lower risk of mortality than the Danish-born, both when looking at crude numbers and after adjustments. This was true both for mortality associated to somatic multimorbidity, psychiatric multimorbidity and the two combined. We did not expect this, since challenges in access to healthcare including language problems, could lead to compliance problems, misunderstandings, and delayed diagnostics and treatment, increasing the risk of mortality. We found no other studies that investigated mortality among migrants with multimorbidity. But, a study published in 2012 by *Norredam* et al. [30]*,* found all-cause mortality to be lower among migrants compared to native Danes, and in 2015 *Byberg* et al. [23] showed lower mortality among all migrant groups with CVD, compared to Danish-born. These results might be explained by less severe multimorbidity burden among the migrant groups or simply less multimorbidity – especially in the family reunification group. The Healthy immigrant effect states, that immigrants represent a selected and healthier subgroup of the population of their country of origin [31]. This could explain both lower multimorbidity and lower mortality among migrants compared to Danish-born who are non-migrants. Other explanations could include the fact that many migrants seek second opinions at health care facilities in their home countries, thus increasing chances of earlier diagnostics, earlier treatment and thereby better survival. Furthermore, the “salmon bias” theory suggests, that elderly and terminally ill migrants tend to emigrate back to their country of origin. As a result, death would not be registered in the immigration country, making it seem like mortality is higher among non-migrants [32]*.*

The study-population included in this study, is relatively young in aspects of multimorbidity which usually increases with age. This is reflected in the somewhat low prevalence of multimorbidity in both our study-group and control-group, compared to the background population of Denmark [3]. When looking at our data, we see that 71,3% of the family reunification immigrants and the matching Danish control group are female, while this only applies to 41,9% of the refugees and their matched Danish controls. Studies have shown higher prevalence of multimorbidity among women compared to men [3, 33, 34]. This is probably also true for our population and could be one of the explanations behind the higher risk of multimorbidity in refugees. But regardless of this explanation, the study shows that refugees are a vulnerable group. When looking at countries of origin, most refugees were from Former Yugoslavia and Eastern Europe, while the family reunification immigrants were mostly from Southern and Eastern Asia. Between these two regions of origin, there is no greater difference in risk of multimorbidity or multimorbidity-related mortality, and from this study we cannot conclude any specific characteristics according to region of origin. Though, this and other studies have shown lower prevalence of multimorbidity and multimorbidity-associated mortality among migrants, we still believe that there is a need for a healthcare system that targets the whole patient in a holistic manner. Most healthcare systems are constructed in a way that targets only single entity diseases, not considering the multimorbid patients and polypharmacy. This creates many problems for both patients and healthcare professionals. We believe in the importance of creating a healthcare system that can contain patients with multimorbidity and other complex issues like language barriers, social issues and so on.

### Methodological strengths and limitations

The main strength of this study is that we could identify and follow a large cohort of migrants, who were matched 1:6 to a Danish-born control-group, making this a unique cohort. Yet our study has certain limitations. First, we only had access to data from the secondary healthcare sector, because the primary healthcare sector in Denmark has no national register of diagnoses. Thus, this study shows multimorbidity among hospitalized patients and ambulatory patients, and is not applicable to the general population of Denmark. Data on medical history and data from Primary care would have made this study more applicable to the general population.

Second, the prevalence of multimorbidity differs according to which method you use and how much history you have [35]. We only had a minimum of two years of history on our cohort, and we used the CCI to assess multimorbidity*.* There is no general agreement on the definition of which chronic diseases that are included in the term multimorbidity. The CCI includes 17 very serious chronic diseases, and people with less severe chronic disease might have been missed in our analysis. Moreover, there is no definition of which psychiatric diagnoses that are chronic, and we chose not to differentiate between chronic and acute psychiatric disease.

Risk of multimorbidity changed somewhat dramatically when we adjusted for mean income. We saw higher risk of multimorbidity among refugees at first, but adjusting for socioeconomic status changed this. Among migrants, income is a difficult cofounder to adjust for, since migrants will always start at a low income and it will take many years for them as a group to get on the same income level as the background population. This is also reflected in Table 1, where the number of refugees in the two highest income groups is very small. We tried to minimize the inequality in annual income, by adjusting for the development in wages, but still it is possible that when adjusting our results for mean income, we made an over-adjustment, making the migrants seem healthier than they really are.

### Future aspects

It could be interesting to investigate if there is a difference in age of debut. Do Danish-born become multimorbid before migrants? Or are migrants diagnosed later than Danish-born? Also, it would be interesting to investigate if Danish-born are more often hospitalized for their chronic diseases, than migrants. Other studies have shown that the advantage in prevalence of multimorbidity among migrants, disappear after a certain amount of years living in Western countries. It would be interesting to see if this pattern is also true for our population. Furthermore, one could investigate if the lower mortality ratio among migrants, found in this study, is related to emigration of critically ill and terminal migrants who wish to die in their home-country (the Salmon bias).

## Conclusion

In this study, we investigated i) risk of multimorbidity and ii) risk of mortality in people with multimorbidity, in non-Western refugees and family reunification immigrants compared to a Danish-born control group. We saw that refugees are an at-risk group for multimorbidity, while family reunification immigrants have lower risk of multimorbidity compared to Danish-born. We found that mortality in those with multimorbidity is lower in all migrant groups compared to Danish-born. Though our results are overall positive for migrants, we still believe that better and more holistic healthcare systems are needed to ensure effective diagnostics and treatment of patients with multimorbidity and other complex issues, like language problems and social issues. There are limited studies in this area, and we believe that this study adds to the existing knowledge. Further research is needed to fully understand the questions raised in this study.

## Abbreviations

AIDS: Acquired Immune Deficiency Syndrome
CCI: Charlson Comorbidity Index
CI: Confidence interval
DNPR: Danish National Patient Registry
DRG: Diagnostic Related Group
HIV: Human Immunodeficiency Virus
HR: Hazard ratio
ICD: International Classification of Diseases
P: Probability value
PIN: Personal Identification Number
WHO: World Health Organization
